# Genetic and Epigenetic Associations with Post-Transplant Diabetes Mellitus

**DOI:** 10.3390/genes15040503

**Published:** 2024-04-17

**Authors:** Zeinab Abdelrahman, Alexander Peter Maxwell, Amy Jayne McKnight

**Affiliations:** 1 Centre for Public Health, Queen’s University of Belfast, Belfast BT12 6BA, UK; z.abdelrahman@qub.ac.uk (Z.A.); a.p.maxwell@qub.ac.uk (A.P.M.); 2 Regional Nephrology Unit, Belfast City Hospital, Belfast BT9 7AB, UK

**Keywords:** association, diabetes, epigenetics, genetics, methylation, NODAT, PTDM, risk, SNP, transplant

## Abstract

Post-transplant diabetes mellitus (PTDM) is a common complication of solid organ transplantation. PTDM prevalence varies due to different diabetes definitions. Consensus guidelines for the diagnosis of PTDM have been published based on random blood glucose levels, glycated hemoglobin (HbA1c), and oral glucose tolerance test (OGTT). The task of diagnosing PTDM continues to pose challenges, given the potential for diabetes to manifest at different time points after transplantation, thus demanding constant clinical vigilance and repeated testing. Interpreting HbA1c levels can be challenging after renal transplantation. Pre-transplant risk factors for PTDM include obesity, sedentary lifestyle, family history of diabetes, ethnicity (e.g., African-Caribbean or South Asian ancestry), and genetic risk factors. Risk factors for PTDM include immunosuppressive drugs, weight gain, hepatitis C, and cytomegalovirus infection. There is also emerging evidence that genetic and epigenetic variation in the organ transplant recipient may influence the risk of developing PTDM. This review outlines many known risk factors for PTDM and details some of the pathways, genetic variants, and epigenetic features associated with PTDM. Improved understanding of established and emerging risk factors may help identify people at risk of developing PTDM and may reduce the risk of developing PTDM or improve the management of this complication of organ transplantation.

## 1. Definition of Post-Transplant Diabetes Mellitus (PTDM)

Hyperglycemia is common following solid organ transplantation in the immediate post-operative period and can be triggered by higher doses of steroid immunosuppression, physiological stress, infection, and enteral or parenteral feeding. This early hyperglycemia may or may not proceed to longer-term hyperglycemia. Early hyperglycemia is best identified by frequent blood glucose monitoring, especially following meals. Historically, post-transplant diabetes mellitus (PTDM [1,2]) has been classified as having random glucose levels greater than 11.1 mmol/L (200 mg/dL), fasting glucose levels ≥ 7.0 mmol/L (126 mg/dL), or the requirement for oral hypoglycemic agents and/or insulin after transplantation. The nomenclature describing this form of diabetes following transplantation has changed over time from steroid diabetes, new-onset diabetes mellitus (NODM), transplant-associated hyperglycemia (TAH), new-onset diabetes after transplantation (NODAT), and more recently, PTDM [2]. In 2014, a panel of experts recommended the adoption of the World Health Organization’s (WHO) definition of diabetes mellitus and impaired glucose tolerance as the basis for defining and diagnosing PTDM [3,4]. These guidelines incorporated the existing random and fasting glucose values into the diagnosis of PTDM and also advocate for the greater use of the oral glucose tolerance test (OGTT) and HbA1c. A two-hour plasma glucose level following a 75 g oral glucose challenge of ≥ 11.1 mmol/L or HbA1c > 48 mmol/mol (6.5%) are diagnostic of PTDM provided the HbA1c values are at least 3 months post-transplant. Many patients who develop PTDM have shown evidence of glucose intolerance or insulin resistance before transplantation [5]. The US Renal Data System (USRDS) data reported that the cumulative incidence of PTDM was 9.1% and 16% at 3 and 12 months post-transplant [5,6,7], rising to 24% by the third year after renal transplantation. PTDM is a recognized early complication of organ transplantation, reflecting the critical importance of transplant-associated factors [5,7].

The reported incidence of PTDM in solid organ transplant recipients varies considerably from up to 30% of renal transplant recipients [8], 27% of heart transplant recipients [9], 17–60% of liver transplant recipients [10], and 40% of lung transplant recipients [11]. The definition of PTDM in transplant recipients also depends on the duration of follow-up, the presence of modifiable and non-modifiable risk factors for diabetes, and the type of organ transplants [12]. For example, in hepatitis C virus-infected liver recipients, the incidence of PTDM has been reported to reach up to 60% [13]. Furthermore, using fasting plasma glucose (FPG) versus OGTT to define diabetes mellitus also changes the prevalence of PTDM [12]. Furthermore, PTDM has been established as an independent risk factor for mortality and is strongly associated with future major cardiovascular events [14].

## 2. Risk Factors for the Development of PTDM

Risk factors for PTDM that are not modifiable include age, race, ethnicity, family history of diabetes mellitus, presence of specific human leukocyte antigens such as HLA A30, B42, and B27, donor–recipient (DR) mismatch, and acute rejection history [12,15,16]. Potentially modifiable risk factors include glucocorticoid-associated PTDM, calcineurin inhibitor-associated PTDM, the interaction between tacrolimus and concomitant hepatitis C infection, the effects of sirolimus on glucose metabolism, obesity, hypertriglyceridemia, hypertension, proteinuria, and hypomagnesemia [12,17].

Glucocorticoids are immunosuppressive agents that have been used in clinical practice since the start of organ transplantation and have the most substantial diabetogenic potential, depending on the dose. Glucocorticoids have detrimental effects on insulin secretion and β-cell apoptosis [18]. Glucocorticoids also increase the prevalence of adiposity and lipolysis, increasing insulin resistance [17]. Additionally, glucocorticoids, associated with insulin resistance, inhibit protein synthesis and accelerate protein degradation in skeletal muscles [19]. Glucocorticoids can increase steatosis in the liver, causing insulin resistance, intensified by increased gluconeogenesis and hyperglycemia [20]. In a study of 25,837 non-diabetic kidney transplant recipients, the cumulative incidence of PTDM within three years of the transplant was 16.2% overall, and 17.7% with maintenance steroids. Patients discharged with steroid-containing immunosuppressive regimens had 42% greater odds of developing PTDM than those not using steroid therapy, indicating a strong association between steroid use and PTDM [21]. Another investigation in kidney transplant recipients explored the relationship between PTDM and genetic variations in the glucocorticoid pathway. An analysis was conducted on the polymorphisms found in genes that are linked to the glucocorticoid receptor (*NR3C1*), P-glycoprotein (*ABCB1*), and glutathione S-transferase P1 (*GSTP1*) [22]. The results indicate that individuals with *GSTP1* genotypes, associated with decreased conjugation capacity, have an elevated risk of developing PTDM. Furthermore, there was a correlation observed between *NR3C1* polymorphism and elevated serum glucose levels following transplantation [22].

For decades, calcineurin inhibitors have been integral components of immunosuppressive regimens for solid organ transplants. However, calcineurin inhibitors have also been strongly associated with acute and chronic nephrotoxicity, electrolyte disorders, metabolic acidosis, neurotoxicity, cardiovascular and metabolic toxicity, and the development of PTDM [23]. Among calcineurin inhibitors, tacrolimus has been associated with a higher incidence of PTDM [23]. Calcineurin inhibitor-induced hypomagnesemia, which is more common with tacrolimus, has also been shown to be an independent risk factor for developing PTDM [24,25]. Several suggested mechanisms for calcineurin inhibitor-induced PTDM include interference with the nuclear factor of activated T cells signaling in pancreatic β-cells and T-cells and decreasing insulin secretion [26]. It was also believed that the high levels of tacrolimus-binding protein 12 in pancreatic β-cells might explain the higher risk of developing PTDM [27]. Sirolimus (rapamycin) is an additional immunosuppressive drug used in transplantation. One of its significant side effects is the increased risk of developing PTDM. The impact of sirolimus on glucose-stimulated insulin secretion is dose-dependent, resulting in marked depletion of calcium in the endoplasmic reticulum and decreased glucose-stimulated mitochondrial Ca^2+^ uptake. This indicates that sirolimus leads to depletion of intracellular Ca^2+^ stores and subsequent reduction in insulin release [28,29]. Sirolimus may also cause β-cell failure due to its mechanism of inhibiting mTORC1-mediated cell proliferation and survival and disturbance of mTORC2 signaling, resulting in impairment in insulin sensitivity [9].

Hepatitis C virus (HCV) is one of the core health issues in kidney and liver transplantation that negatively affects patient and graft survival. HCV is associated with a higher risk for PTDM [30]. The mechanisms underlying the diabetogenic effect of HCV are complicated but likely to involve increased insulin resistance caused by inhibitory actions of the virus on the insulin regulatory pathways in the liver, resulting in increased morbidity and mortality in liver and kidney transplant recipients [30]. CMV infection also remains one of the major causes of morbidity and mortality after renal transplantation [31]. Both asymptomatic CMV infection and CMV disease are independent risk factors for the early development of PTDM. Generally, transplant recipients with active CMV infection are characterized by a notably lower insulin secretion compared to patients without CMV infection [31]. 

Early post-transplant hyperglycemia, within the first few weeks, is common and associated with higher doses of immunosuppression, infections, and stresses related to the peri-operative period [32]. This early hyperglycemia is usually transient but may still need insulin therapy to manage the situation until normoglycemia returns. If hyperglycemia does persist, it is usually possible to reduce or discontinue insulin whilst introducing oral hypoglycemic medication. If PTDM has developed then it is best managed with the same standards and treatment targets that have been established for type 2 diabetes [32].

## 3. Transplant Genetics, Epigenetics, and Their Association with PTDM

Most organ transplants are allografts, which has prompted researchers to consider the consequences of genetic variations between donor organs and recipients’ genomes [33]. Significant genomic differences between donor and recipient can include 3.5 million to 10 million genetic variants and additional epigenetic modifications, reflecting different factors such as the background ethnicity, lifestyles, and geographical region of donors and recipients [34]. It is challenging to unravel the interplay of these underlying genomic factors against the background of the known modifiable and non-modifiable risk factors for PTDM. It may be possible to explore a variety of ‘omics’-based approaches to determine which genomic factors predispose to PTDM and to explore if any of these genetic variants or epigenetic features will help discover novel drug targets or lead to more personalized immunosuppressive treatments. Pre-transplant genetic analysis of donors can detect organs that are more vulnerable to graft damage and aid in decreasing the likelihood of graft rejection [35], although the implementation of this in clinical settings poses challenges. In contrast, identifying recipients at a higher risk of adverse outcomes, such as PTDM, enables more intensive monitoring for hyperglycemia and appropriately modified immunosuppressive therapy, e.g., steroid-sparing, to reduce the risk of developing PTDM [36].

Graft rejection, whether hyperacute, acute, or chronic, can occur through two primary pathways of processing and presenting donor antigens to recipient immune cells: direct and indirect [36]. Following transplantation, naive T cells become activated and differentiate into pro-inflammatory or anti-inflammatory subtypes [36]. This activation may lead to distinct hyperimmune response patterns that ultimately result in the destruction of the graft, mediated by cytotoxic T cells or antibodies [36]. Hyperacute rejection typically manifests within minutes to hours post-transplantation and is driven by the humoral immune response [36]. Pre-existing antibodies against donor antigens trigger the activation of the complement system, contributing to graft destruction. In contrast, acute and chronic rejection can occur days, months, or even years after transplantation, often as a result of T cell-mediated or antibody-mediated mechanisms [36]. However, chronic graft injury can also arise from non-immune factors such as infections, ischemia, aging, and drug toxicity [36]. 

Identifying associations between genetic polymorphisms and their regulatory regions and transplant outcomes has been one of the main concerns in transplantation [37]. In the human genome, HLA class I and class II genes exhibit the highest degree of polymorphism. Depending on the number of HLA mismatches, graft survival probability decreases. This is why HLA typing is essential for identifying incompatibilities and allows the suitable matching of donor and recipient pairs [38]. It is worth noting that even patients who undergo HLA-identical transplants are susceptible to acute or chronic rejection, indicating the involvement of other factors in alloimmunities such as killer-cell immunoglobulin-like receptors (KIRs), MHC class I polypeptide-related sequence A (MICA), and minor histocompatibility antigens (mHAs). The sufficient antigenicity of minor histocompatibility antigens (mHAs) allows for the induction of a directed immune response against the non-self-antigen following transplantation [39]. Although the epidemiological data indicates a notable impact on long-term graft survival, the precise significance of these minor histocompatibility antigens in solid organ transplantation is not fully comprehended [39]. Additionally, researchers have identified many genome-wide genetic polymorphisms among individuals with several thousand non-synonymous genetic variants causing alterations in the amino acid sequences of proteins [40]. For example, mismatches between the recipient KIRs and donor ligands in kidney transplantation were associated with reduced long-term graft survival despite donor-recipient pairs being matched for HLA-A, HLA-B, and HLA-DR alleles [41].

There is some evidence of a genetic predisposition to PTDM, including established associations with the alleles HLA A28, A30, B27, and Bw42. Furthermore, a report has emphasized the association of *TCF7L2* rs7903146 and *SLC30A8* rs13266634 polymorphisms with a higher risk of PTDM [42,43,44,45]. Another study investigated the association of genetics with PTDM in a white UK renal transplant population; 26 SNPs were associated with PTDM, such as *ATP5F1P6* rs1048482, *DNAJC16* rs7533125, and *CELA2B* rs2861484 [46]. Other associations of specific gene variants with PTDM include *KCNQ1* SNP rs2237895 [47], *NFATc4* [48], *HNF1B* [49], adiponectin rs1501299 [50], mitochondrial haplotype H [51], *CDKAL1* rs10946398 [51], *KCNJ11* rs5219 and rs5215 SNPs [52], and *NPPA* rs198372 [53]. While these associations are interesting, the findings have not been independently replicated in different populations. Further prospective studies are needed to establish if these genetic variants are independent risk factors for PTDM. 

Additionally, there are multiple SNPs previously reported to be associated with PTDM, such as *CAPN10* rs5030952, *CCL5* (rs2107538, rs2280789, rs3817655), *ENPP1* rs1044498a, *GPX1* rs1050450, *HNF1A* (rs1169288a, rs1800574a), *HNF4A* (rs2144908, rs1884614, rs1800961), *IFNγ* rs2430561, *IL-17E* rs1124053, *IL-17RA* (rs2229151, rs4819554), *IL-17RB* (rs1043261, rs1025689), *IL-1B* rs3136558, *IL-2* (rs2069762, rs2069763), *IL-4* (rs2243250, rs2070874), *IL-6* rs1800795, *IL-7R* (rs1494558, rs2172749), *IRS1* rs1801278, *PPARG* rs1801282a, *PPARGC1* rs8192678a, and *SUR1*(rs1799854a, rs1801261a) [46] (Table 1).

Beta-cell (β-cell) dysfunction is considered to be the main contributing factor for PTDM development. For example, *KCNQ1* encodes a subunit of a voltage-gated K+ channel in pancreatic β-cells, and *KCNQ1* genetic variants are associated with β-cell dysfunction in renal transplant recipients. The overexpression of *KCNQ1* in the pancreatic β-cell has been associated with increased K+ current density, causing changes in the pancreatic cell membrane action potential [59]. Moreover, *KCNQ1* overexpression is associated with impaired glucose-stimulated insulin secretion. Also, allelic mutation of *KCNQ1* results in the upregulation of *CDKN1C*, which reduces pancreatic β-cell mass [59]. Of interest, individuals with *HNF1B*-associated renal disease are at increased risk of developing PTDM, and analysis of the *HNF1B* gene mutations might be useful in predicting PTDM in potential renal transplant recipients [49].

Increased expression of *IL-17E*, *IL-7R*, *IL-17R*, and *IL-17RB* has been identified as another mechanism underlying β-cell dysfunction and their reported association with PTDM in renal transplant patients [72]. Moreover, the AG heterozygous variant of the *MBL2* gene rs2232365 has a higher risk of PTDM than the AA variant [54]. 

In 2014, the first genome and epigenome-wide association study was conducted to investigate variants associated with PTDM [78]. The study discovered eight novel SNPs associated with PTDM, including rs10484821, rs7533125, rs2861484, rs11580170, rs2020902, rs1836882, rs198372, and rs4394754 [78]. Most of the SNPs identified in this study are implicated in β-cell apoptosis, suggesting that β-cell stress due to post-transplant hyperglycemia could be critical in the pathogenesis of PTDM [78]. 

There are several risk factors for PTDM where epigenetics may play an important role. These include epigenetic features associated with non-modifiable factors such as a person’s age [79] and ethnicity [80], as well as epigenetic associations with intrinsic and extrinsic factors. DNA methylation is strongly associated with kidney disease [81] and kidney transplantation [82], as well as socioeconomic position [83], BMI [84], cardiovascular events [85], and premature mortality [86]. Understanding more about the role of DNA methylation in PDTM may help guide personalized management. For example, a recent study observed that DNA methylation not only predicted weight loss but also helped target individual diets that were most appropriate to support individuals losing weight [87].

Epigenetic modifications have been reported to be associated with renal transplant outcomes [88]. It is likely that the dynamic interactions between the recipient’s immune system, immunosuppressive therapy, and donor organ are influenced in part by epigenetic mechanisms. Immunological tolerance is associated with demethylation of immune system genes regulating B-cell and T-cell activation and chronic rejection associated with altered DNA methylation (mainly hypomethylation) in genes controlling ubiquitination pathways involved in protein degradation that regulate antigen presentation as well as B-cell receptor and T-cell receptor signaling [88,89,90]. Likewise, altered DNA methylation can control *IL-2* expression, which mediates the activation of T cells [91]. Notably, DNA methylation profiles are crucial for the stability of Treg cells and maintaining the steady expression of *FOXP3* protein [92], and higher *FOXP3* protein levels were associated with worse allograft outcomes and diabetic pathogenesis [93,94].

There are relatively few studies of epigenetic features associated with PTDM. Most of the reports are cross-sectional case-control studies comparing transplant recipients with PTDM with age and sex-matched controls. One such study reported that there were similarities between the DNA methylome in adipose tissue of renal transplant recipients with PTDM and the DNA methylome of individuals with type 2 diabetes [95]. Another study established an epigenetic profile reference for kidney transplant patients and identified potential markers associated with glomerular filtration rates and diabetic kidney disease, such as cg23597162 within *JAZF1* and cg17944885 [82]. A prospective study of individuals with end-stage renal disease who have DNA samples taken prior to transplant and post-transplant would help to discover the epigenetic profiles that predict the development of PTDM (and other important renal transplant outcomes).

From a transcriptomic perspective, studies have been conducted to identify marker genes associated with graft rejection. For example, a study investigated the association of immune-related genes with graft rejection after kidney transplantation [96]. Several identified genes were strongly correlated with graft rejection, including *CXCL11*, *CCL4*, *CXCL10*, *IDO1*, and *GBP2* [96]. *CXCL10* and *CXCL11* are crucial in many inflammatory diseases [97]. They were strongly associated with pro-inflammatory mediators, such as *IL-1β*, *IL-2*, *IL-6*, and *TNF-*α, that are integral to various inflammatory disorders such as coronary heart disease, insulin resistance, and diabetes [97,98]. Furthermore, *IDO1* variants are also strongly associated with type 2 diabetes. Several reports indicate that IDO dysregulation is implicated in insulin resistance [99,100,101]. *GBP2*, a family member of IFN-stimulated GTPases expressed in human cells, is highly responsive to IFN stimulation and regulates cellular proliferation, tissue invasion, and angiogenesis [102]. Moreover, GBP2 has an essential role in immunity, infection, inflammation, and metabolic syndromes and could have a role in insulin resistance [103]. In a study of transcriptomic activity in post-transplant kidney allografts [104], the authors identified strongly correlated gene clusters associated with fibrosis (e.g., *COL1A1*, *DPT*, and *MMP7*), B-cell activity, and immunity (e.g., *CD52*, *CXCL10*, and *CCL21*); several of these genes have been associated with insulin resistance and diabetes [104,105,106,107,108].

MicroRNAs are involved as transcriptional regulators in various biological processes, and extensive research efforts have identified associations between transplantation and specific microRNAs (e.g., miRNA-21, miRNA-146, miRNA-155, miRNA-199, and miRNA-214) [109]. However, the role of miRNAs in PTDM remains poorly understood. Identifying miRNA signatures helps reveal molecular pathways involved in pre/post-transplantation responses [109]. For example, in kidney injury, microRNA-21 becomes activated and is also involved in type 2 diabetes mellitus complications [110]. miRNA-21 stimulates renal fibrosis in diabetic nephropathy and regulates adipogenic differentiation through TGF-β signaling [111]. Another example is the higher expression of miRNA-146 and miRNA-155 in the renal tissue obtained from kidney transplant recipients, and studies have confirmed the strong association of these miRNAs with diabetes [109]. One study indicates that miRNA-155 and miRNA-146a expression increased more than fivefold in the kidney samples of diabetic nephropathy patients [112]. miRNA-155 regulates insulin sensitivity, and its dysregulation contributes to developing diabetes mellitus complications [113]. Downregulation of miRNA-146a may be associated with a predisposition to type 2 diabetes [114]. Additionally, miRNA-199 was found to affect pancreatic β-cell function negatively, and its expression is highly increased in the plasma of people with diabetes [115]. miRNA-214 is involved in skeleton formation and cancer [109], and microarray analysis has shown significant overexpression of miRNA-214 in the renal cortex of diabetic mice, which suggests that it may be a therapeutic target for diabetic nephropathy [116].

## 4. Pathways Associated with PTDM

**Figure 1:**
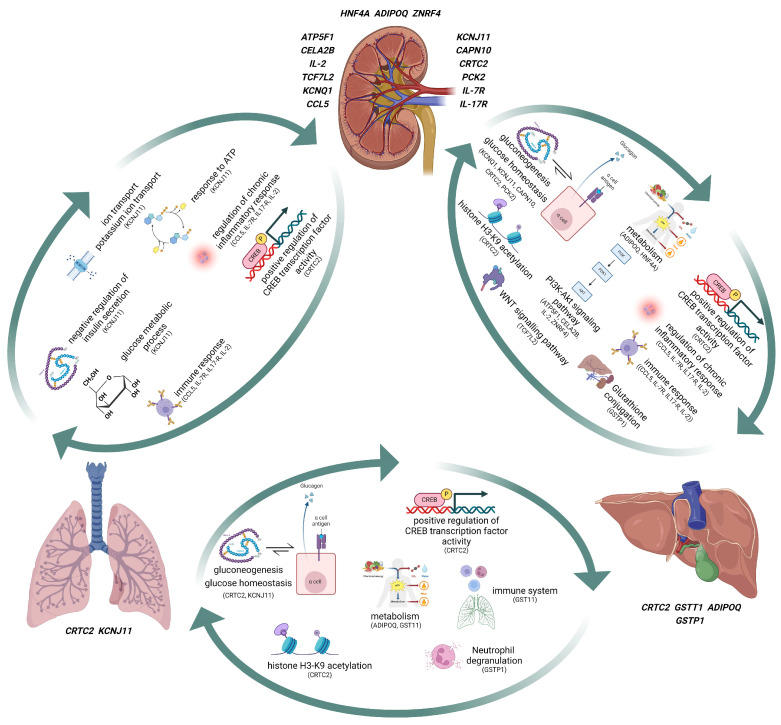
Biological pathways of genes associated with PTDM in lungs, liver, and kidneys.

PTDM has been associated with pathways involved in insulin sensitivity and/or insulin deficiency as well as pathways linked to hyperglycemia and vascular complications. Glucose production in the liver results from a combination of gluconeogenesis and glycogenolysis [117]. Insulin is a crucial hormone regulator of gluconeogenesis, and the indirect effect of insulin can suppress gluconeogenesis in several tissues and cell types, such as pancreatic cells [117]. Insulin in pancreatic α cells inhibits glucagon secretion, which can, in turn, suppress hepatic glucose production by decreasing glucagon signaling in the liver [118]. Increased hepatic glucose production increases glucose release into the blood, which can cause hyperglycemia [117], and in people with diabetes, gluconeogenesis has been identified as the main source of glucose production [119]. Insulin may modify hepatic gluconeogenesis by transcribing gluconeogenic control genes, including *PCK1* and *G6PC*. Analysis of liver biopsies from people with diabetes demonstrated no elevation of *PCK1* and *G6PC* [120]. In contrast, a study on patients revealed a direct link between insulin resistance and the mRNA levels of *PCK1*, *G6PC*, and *FOXO1* [121]. Since gluconeogenesis is a primary factor in hepatic glucose production in people with diabetes and insulin impacts gluconeogenesis through transcription, identifying the insulin signaling pathways that affect gluconeogenic gene transcription and gluconeogenesis can contribute to comprehending PTDM pathophysiology (Figure 1).

Insulin binding to the insulin receptor activates tyrosine kinase, which initiates signaling. Activation of this process results in the phosphorylation of multiple intracellular substrates, such as the PI3K/AKT and MAPK signaling pathways [122]. The PI3K/AKT and MAPK signaling pathways can be compromised in people with diabetes, which can cause enhanced hepatic glucose production and decreased insulin sensitivity, implying a major role for these substrates in maintaining glucose balance [123] (Figure 1). On the other hand, hepatic CREB’s role in diabetes is amplified transcriptionally, resulting in hyperglycemia and insulin resistance [124]. The transcription factor CREB has been observed when it has decreased expression levels in the liver of mice, leading to lower blood glucose levels and lower expression of gluconeogenic genes; this demonstrates the importance of CREB in hepatic gluconeogenesis [125]. In the absence of insulin, the CREB complex activates and interacts with RNA polymerase II to augment the expression of the glucose-production genes. Furthermore, the increased phosphorylation of CREB due to elevated glucose, insulin, and L-glutamate levels illustrates the crucial role of CREB in diabetic complications [126] (Figure 1).

Inflammation plays a prominent role in the pathophysiology of diabetes and its related metabolic disorders, therefore targeting inflammation may help in the prevention and control of diabetes. Inflammation can disrupt normal insulin signaling [127] (Figure 1). Both acute and chronic inflammation are associated with raised levels of cytokines and insulin resistance [128]. As diabetes progresses, there are alterations in immune B cell profiles and the patterns of CD8+ T cell migration [129]. Macrophages also have a fundamental role in inflammation, as they can secrete cytokines like interleukin 1 β and TNF-α, and generate reactive oxygen species (ROS) [130]. Studies have suggested a link between inflammation of the pancreatic β-cells and PTDM, a likely association between heightened innate immune system activity and the emergence of PTDM, and the involvement of TNF [67,72,131,132]. One study demonstrated an association between augmented IL6 levels and PTDM, and elevated IL6 and TNF receptor II concentrations are correlated with a greater probability of graft failure and mortality [133], suggesting a role of inflammation in PTDM pathogenesis. 

Wnt signaling is of major importance in embryonic development and the pathogenesis of cancer and has been shown to be a key controller of a variety of illnesses, including prostate cancer and type 2 diabetes [134]. The first association between the Wnt signaling pathways and diabetes identified a genetic polymorphism of the *TCF7l2* gene, which encodes a vital transcription factor TCF4 in the Wnt signaling pathways [135]. Additionally, interference in Wnt signaling pathways is involved in the development of diabetes, as it affects the proliferation and differentiation of pancreatic β-cells and the secretion of insulin [136]. These findings imply that Wnt could significantly affect the development and progression of diabetes (Figure 1). CREB functions as a transcriptional factor, prompting the expression of gluconeogenesis enzymes [137]. The transcriptional activity of hepatic CREB is amplified in diabetes, resulting in high blood sugar and a lack of responsiveness to insulin [124] (Figure 1). Obesity, associated with type 2 diabetes, can lead to an increased expression of hepatic proteins of CREB, which in turn can cause hyperglycemia, hyperinsulinemia, and hyperglucagonemia [124]. Evidence for CREB’s involvement in the progression of diabetic complications, including diabetic coronary atherosclerosis, has been established [124].

Furthermore, CREB phosphorylation is augmented by high glucose and insulin concentrations, suggesting that enhanced CREB phosphorylation may play a role in neurodegenerative diseases and diabetic complications [126]. Histone acetylation has also been linked to diabetic complications, such as diabetic retinopathy [138] (Figure 1). Recent evidence indicates that histone modification appears to initiate oxidative stress, inflammation, and fibrosis in the diabetic kidney [139]. Research conducted on diabetic mice showed that early glomerulosclerosis was linked to diminished H3K9 and H3K23 histone acetylation [140]. Further investigations revealed that the acetylation levels of H3K9, H3K18, and H3K23 were elevated in the kidneys of diabetic mice [141,142]. It was seen that in hyperglycemia, increased H3K9 and H3K18 acetylation levels in the mesangial cells correlated with the production of inflammatory mediators [141]. It is unclear if inhibitors of CREB phosphorylation or histone modification could prevent or delay the development of PTDM. 

Although there are interesting parallels between the molecular pathogenesis of type 2 diabetes and PTDM, there are unique triggers to PTDM related to the transplant itself and post-transplant treatment. The pathogenesis of PTDM is expected to entail multifaceted interactions among several factors, including the degree of HLA mismatch between kidney donor and transplant recipient, immunosuppressive medications, and genetic predisposition to diabetes. Consequently, it is vital to conduct targeted research that centers on PTDM to discover the pivotal pathways in its development and progression.

## 5. Conclusions

A thorough comprehension of PTDM necessitates an exploration of its intricate interactions across genetic, molecular, and clinical domains. The primary focus of targeted research should be on the identification of key pathways, validation of genetic associations, and investigation of therapeutic interventions aimed at minimizing the risk of PTDM, ultimately leading to improved outcomes for organ transplant recipients. The significance of insulin resistance and impaired insulin secretion prior to or following transplantation has been highlighted in recent studies investigating the mechanism of PTDM. This emphasizes the necessity of promptly detecting and managing insulin resistance in transplant recipients. PTDM clinical management still contains numerous areas of ambiguity, while the debate over the most suitable screening strategy remains unsettled. The primary concern that needs to be addressed is how to effectively reduce the risk of PTDM in transplant recipients. It is imperative to conduct additional research and development to enhance screening and management strategies, thereby enhancing the quality of life for transplant recipients.

## Figures and Tables

**Table 1:** Reported gene single nucleotide polymorphisms (SNPs) and their association with post-transplant diabetes mellitus (PTDM). ADA = American Diabetes Association; FBG = fasting blood glucose; HbA1c = glycated hemoglobin.

1st Author and Year	Type of Study	PTDM Definition	Population Characteristics	Outcome SNP to the Nearest Gene
Guad, 2020 [54]	29 cases of PTDM after kidney transplantation and 139 controls	PTDM was defined based on the ADA guidelines: FBG ≥ 126 mg/dL and not on insulin or oral anti-diabetic drugs at any point of the follow-up period within one year of renal transplant. Controls have been defined as patients who did not develop PTDM during the follow-up period. There is no evidence of kidney or diabetic damage among controls.	Mixed-race patients without a history of diabetes mellitus before renal transplant and had attended their follow-up at one month, three months, six months, and one year. Two SNPs of *IL7R* (rs1494558) and *MBL2* (rs2232365) were genotyped for the study.	rs1494558 (*IL7R*); OR:3.1, 95% CI: 1.3–7.9, *p =* 0.01 rs2232365 (*MBL2*); OR:2.6, 95% CI: 1.1–6.2, *p =* 0.04
Elbahr, 2020 [55]	64 cases of PTDM after liver transplantation and 94 controls	PTDM was defined based on the ADA guidelines: the requirement of insulin or hypoglycaemic agents, hemoglobin A1c (HbA1c) ≥ 6.5% or FBG ≥ 126 mg/dL on two separate occasions, or random blood glucose ≥ 200 mg/dL associated with diabetic symptoms. Controls have been defined as patients who did not develop PTDM during the follow-up period. There is no evidence of liver or diabetic damage among controls.	Patients aged ≥ 18 and ≤ 45 years underwent primary living-related donor liver transplantation and survived > 1 year post-transplant. Follow-up time > 1 year post-transplant. rs738409 SNP of the *PNPLA3* gene was genotyped for the study.	rs738409 (*PNPLA3*); OR:5.5, 95% CI: 2.4–12.7, *p =* 0.00
Van der Burgh, 2019 [56]	29 cases of PTDM after kidney transplantation and 138 controls.	PTDM was defined according to the ADA guidelines. Patients who used anti-diabetic drugs after transplantation were also considered to have PTDM. Controls have been defined as patients who did not develop PTDM during the follow-up period. There is no evidence of kidney or diabetic damage among controls.	Mixed-race patients aged ≥ 18 years who received a single-organ, blood group ABO-compatible kidney from a living donor without a history of diabetes mellitus before renal transplantation. Follow-up period at 1, 3, 6, and 12 months after kidney transplant. Three SNPs of the *HNF1β* allele (rs752010, rs4430796, and rs7501939) were genotyped for the study.	rs752010 (*HNF1β*); OR:2.6, 95% CI: 1.1–6.2, *p =* 0.04
Mota-Zamorano, 2019 [57]	57 cases of PTDM after kidney transplantation and 258 controls	PTDM was defined according to the ADA guidelines: two FBG ≥ 7.0 mmol/L or symptoms of diabetes plus casual plasma glucose concentrations ≥ 11.1 mmol/L throughout the first year. Controls have been defined as patients who did not develop PTDM during the follow-up period. There is evidence of kidney or diabetic damage among controls.	Caucasian recipients without a history of diabetes mellitus received a single kidney from deceased donors—a follow-up period of one week, one month, five months, and one year after the kidney transplant. Three SNPs of the *LEPR* gene (rs1137100, rs1137101, and rs1805094) were genotyped for the study.	rs1137101 (*LEPR*); OR:3.2, 95% CI:1.4–7.9, *p =* 0.009
Musavi, 2019 [58]	52 cases of PTDM after liver transplantation and 54 controls.	PTDM was defined according to the ADA guidelines: FBG level ≥ 7 mmol/L (≥ 126 mg/dL) or a non-FBG level ≥ 11.1 mmol/L (≥ 200 mg/dL), confirmed on at least two occasions, or taking anti-diabetic drugs for > 1 month after transplant. Controls have been defined as patients who did not develop PTDM during the follow-up period and with no history of glucose intolerance. There is no evidence of liver or diabetic damage among controls.	Patients with no previous diagnosis of diabetes, pretransplant fasting plasma glucose level < 5.5 mmol/L, and undergoing follow-up for ≥ 10 months. *GSTM1*, *GSTP1*, and *GSTT1* genetic polymorphisms were genotyped for the study.	AG genotype (*GSTT1*); OR:15.8, 95% CI:4.5–60.2, *p =* 0.00 AG + AA genotype (*GSTP1*); OR:6.2, 95% CI: 1.2–29.6, *p =* 0.002
Hwang, 2019 [59]	254 cases of PTDM after kidney transplantation and 848 controls.	PTDM was defined according to the ADA guidelines: FBG was higher than 126 mg/dL six months after transplantation or when insulin or oral anti-diabetic agents were required for treatment. Controls have been defined as patients who did not meet PTDM criteria during the follow-up period. There is no evidence of kidney or diabetic damage among controls.	Asian patients with no previous diagnosis of diabetes, follow-up period for more than one year, and functioning graft at one-year follow-up. A total of 17 SNPs were genotyped for the study.	rs2237892 (*KCNQ1*); OR:0.63, 95% CI: 0.51–0.79, *p* < 0.0001
Zhang, 2018 [60]	17 cases of PTDM after kidney transplantation and 112 controls.	PTDM was defined according to the ADA guidelines: HbA1c continuously > 6.5%, FBG of > 126 mg/dL (7.0 nmol/L), or requiring insulin and/or oral anti-diabetic agents for > 3 months. Controls have been defined as patients who did not develop PTDM during the follow-up period. There is evidence of kidney or diabetic damage among controls.	Chinese Han ethnic patients aged > 18 with primary renal transplantation and no history of type 2 diabetes before transplantation. The follow-up period was one year. The following SNPs, including *CYP3A5* rs776741, rs776746, rs15524, *CYP24A1* rs2296241, and *PPARG* rs1801282, were genotyped for the study.	rs2296241 (*CYP24A1*); OR:7.1, 95% CI: 1.3–38.4, *p =* 0.02
Daohua, 2018 [61]	57 cases of PTDM after kidney transplantation and 112 controls	Patients were diagnosed with PTDM by FBG ≥ 7.0 mmol/L, two h post-load glucose ≥ 11.1 mmol/L during OGTT, or required insulin and/or oral anti-diabetic drugs for more than three months after transplantation. Controls have been a group of type 2 diabetic mellitus patients and healthy volunteers. There is evidence of kidney or diabetic damage among controls.	Chinese Han ethnic patients undergo the first renal transplantation and maintain renal allograft function at least one year after the transplant. Genetic polymorphisms of *CYP3A4*, *CYP3A5*, *ABCC8*, and *GCK* were genotyped for the study.	* 18B (*CYP3A4*); OR:2.1, 95% CI: 1.1–4.2, *p =* 0.04 G-30A (*GSK*); OR:2.5, 95% CI: 1.1–5.8, *p =* 0.03
Yokoyama, 2018 [62]	11 cases of PTDM after kidney transplantation and 27 controls.	PTDM was defined as having FBG > 140 mg/dL at 120 min in the 75-g OGTT one year after transplantation. Controls have been defined as patients with <139 mg/dL plasma glucose levels. There is evidence of kidney or diabetic damage among controls.	Patients aged > 18 years with no history of diabetes. The follow-up period was one year. A total of 8 SNPs in 7 genes were genotyped for the study, rs1499821 and rs5398 in *SLC2A2*, rs4982856 in *PCK2*, rs4402960, rs10811661, rs1111875, rs13266634, and rs7756992 in *IGF2BP2*, *CDKN2A/B*, *HHEX*, *SLC30A8*, and *CDKAL1*.	rs4982856 (*PCK2*); OR:10.1, 95% CI: 2.1–48.8, *p =* 0.003
Quteineh, 2018 [63]	152 cases of PTDM after solid organ transplantation and 544 controls (kidney 69.1%, liver 15.5%, lung 9.2%, and heart 6.2%).	PTDM was diagnosed in instances where patients required antidiabetic treatments post-transplantation or if a new metabolic event was reported in the case report forms.	The study excluded individuals younger than 18 years old and individuals who had undergone multiple organ transplantation. This study involved the selection of 287 SNPs located within 158 genes that are known to be involved in the immune response to infectious pathogens or inflammation.	SP110 rs2114592C > T; OR: 8.90, 95% CI: 1.97–40.0, *p =* 0.04
Chao, 2017 [50]	75 cases of PTDM after liver transplantation and 181 controls.	PTDM has been defined as having FBG of at least 7.0 mmol/L (126 mg/dL) or a non-fasting FBG of at least 11.1 mmol/L (200 mg/dL) on at least two occasions or requiring anti-diabetic drugs beyond the first month after transplantation. Controls have been defined as patients who did not develop PTDM. There is evidence of liver or diabetic damage among controls.	Patients aged > 18 years, with no history of diabetes, transplantation, or acute graft rejection, and a follow-up period > 6 months. Twelve SNPs were genotyped for this study: *ADIPOQ* rs1501299, *ADIPOQ* rs822396, *ADIPOR2* rs767870, *TLR4* rs1927907, *CCL5* rs2107538, *CCL5* rs2280789, *CYP3A5* rs776746, *PPARA* rs4823613, *ACE* rs4291, *HSD11B1* rs4844880, *KCNJ11* rs5219 and *KCNQ1* rs2237892.	rs1501299 (*ADIPOQ*); OR:1.88, 95% CI: 1.1–3.4, *p =* 0.03
Quteineh, 2017 [64]	**Lung:** 7 cases of PTDM after lung transplantation and 10 controls **Kidney:** 27 cases of PTDM after kidney transplantation and 75 controls **Liver:** 11 cases of PTDM after liver transplantation and 26 controls.	PTDM was defined as requiring anti-diabetic treatment (either insulin or oral anti-diabetic agents) for at least six months following transplantation or having several abnormal glucose profiles during the follow-up period that fulfill the criteria given by the WHO and ADA consensuses, including FBG ≥ 7.0 mmol/L (in ≥two occasions) or 2 h plasma glucose ≥ 11.1 mmol/L during OGTT. Controls have been defined as patients who did not develop PTDM. There is evidence of organ or diabetic damage among controls.	Patients aged > 18 years with functional graft for more than 12 months after transplantation, with no previous history of diabetes or previous transplantation. The follow-up period was five years. Two SNPs within the *CRTC2* gene (rs8450 and rs12117078) were genotyped for the study.	rs8450 (*CRTC2*); OR:6.9, 95% CI: 1.5–31.3, *p =* 0.02
Yalin, 2017 [65]	52 cases of PTDM after kidney transplantation and 257 controls.	PTDM was defined according to the American Diabetes Association guidelines: 2 h Postprandial Plasma Glucose levels obtained from OGTT in patients without overt hyperglycemia. Controls have been defined as patients with renal transplantation and normal glucose metabolism after transplantation.	Patients with no previous history of diabetes and with a functional graft. The follow-up period was > 3 years. rs1050450 of the *GPX1* and rs1805127 of the *KCNJ11* were genotyped for the study.	rs1050450 (*GPX1*); OR:29.9, 95% CI: 2.8–314.5, *p =* 0.01
Ong, 2016 [66]	52 cases of PTDM after kidney transplantation and 257 controls.	PTDM was defined as having HbA1c > 6.5%, FBG levels > 7.0 mmol/L (126 mg/dL), or requiring anti-diabetic medication or insulin for > 3 months. Controls have been defined as patients who did not develop PTDM. There is evidence of kidney or diabetic damage among controls.	Patients were included if they with normal fasting glucose levels (FPG < 100 mg/dL) and HbA1c levels (< 6%) before transplantation. The mean follow-up duration was 90 months. Eleven SNPs within the *MMP1*, *MMP2*, and *MMP3* genes were genotyped for the study.	rs1132896 (*MMP2*); OR:3.8, 95% CI: 1.2–12.2, *p =* 0.03 rs243849 (*MMP2*); OR:0.32, 95% CI: 0.13–0.75, *p =* 0.009
Kim, 2016 [67]	52 cases of PTDM after kidney transplantation and 257 controls.	PTDM was defined according to ADA guidelines (it is not specified which criteria were used for diagnosing diabetes). Controls have been defined as patients who did not develop PTDM. There is evidence of kidney or diabetic damage among controls.	Patients aged > 18 years with no history of diabetes. The follow-up duration was one year after transplantation. A total of 6 TLR genes (*TLR2* rs3804099, *TLR2* rs3804100, *TLR4* rs1927914, *TLR6* rs3775073, *TLR6* rs3821985, and *TLR6* rs1039559) were genotyped for the study.	rs1927914 (*TLR4*); OR:0.55, 95% CI: 0.35–0.86, *p =* 0.008 rs3775073 (*TLR6*); OR:0.57, 95% CI: 0.35–0.94, *p =* 0.02 rs3821985 (*TLR6*); OR:0.57, 95% CI: 0.35–0.94, *p =* 0.02 rs1039559 (*TLR6*); OR:2.0, 95% CI: 1.3–3.2, *p =* 0.002
Khan, 2015 [42]	42 cases of PTDM after kidney transplantation and 98 controls.	PTDM was defined according to ADA guidelines: FBG level of more than 126 mg/dL. Controls have been defined as patients who did not develop PTDM. There is no evidence of kidney or diabetic damage among controls.	Asian Indian patients who developed PTDM after transplantation. The follow-up period was not specified. *TCF7L2* (rs7903146) and *SLC30A8* (rs13266634) were genotyped for the study.	rs7903146 (*TCF7L2*); T vs. C: OR:2.6, 95% CI:1.2–5.6, *p* = 0.01 rs13266634 (*SLC30A8*); T vs. C: OR: 2.0, 95% CI: 1.1–3.4; *p* = 0.01
Romanowski, 2015 [68]	23 cases of PTDM after kidney transplantation and 146 controls.	PTDM was defined as hemoglobin A1c continuously over 6.5%, FBG ≥ 7.0 mmol/L, or requiring treatment with oral anti-diabetic agents or insulin for more than three months after transplantation. Controls have been defined as patients who did not develop PTDM. There is evidence of kidney or diabetic damage among controls.	Patients with no history of diabetes mellitus before transplantation and with a functional graft. Patients deceased within 1-month post-transplant were excluded. The follow-up period was three years. *ADIPOQ* rs266729, rs1501299, and *LEP* rs2167270 SNPs were genotyped for the study.	rs2167270 (*LEP*); OR:4.0, 95% CI:1.5–10.5, *p =* 0.002
Tavira, 2014 [51]	115 cases of PTDM after kidney transplantation and 197 controls.	PTDM was defined as FBG > 125 mg/dL (7.0 mmol/L) after three consecutive measurements. Controls have been defined as patients who did not develop PTDM. There is evidence of kidney or diabetic damage among controls.	Caucasian patients with no history of diabetes mellitus before transplantation. The follow-up duration was one year after transplantation. mtDNA polymorphisms were genotyped for the study.	mitochondrial haplotype H; OR:1.82, 95% CI:1.1–2.9, *p =* 0.01
McCaughan, 2014 [46]	57 cases of PTDM after kidney transplantation and 370 controls.	PTDM was defined as a new requirement for oral anti-diabetic agents or insulin to manage hyperglycemia after transplantation. Controls have been defined as patients who did not develop PTDM and did not develop a requirement for oral anti-diabetic therapy or insulin during the follow-up period. There is evidence of kidney or diabetic damage among controls.	99% of white patients aged ≥ 16 years. The median follow-up time was 12.2 years (0–26.0 years). GWAS analysis of 561,233 single-nucleotide polymorphisms (SNPs) were genotyped for the study.	rs10484821 (*ATP5F1P6*); OR:3.5, 95% CI: 2.1–5.8, *p* = 1.5 × 10^−7^ rs7533125 (*DNAJC16*); OR:2.4, 95% CI: 1.5–3.6, *p* = 0.001 rs2861484 (*CELA2B*); OR:2.4, 95% CI: 1.5–3.7, *p* = 0.001 rs11580170 (*AGMAT*); OR:2.2, 95% CI: 1.4–3.4, *p* < 0.00 rs2020902 (*CASP9*); OR:2.3, 95% CI: 1.5–3.6, *p* < 0.001 rs1836882 (*NOX4*); OR:2.7, 95% CI: 1.5–3.8, *p* < 0.001 rs198372 (*NPPA*); OR:2.5, 95% CI:1.5–4.2, *p* < 0.001 rs4394754 (*INPP5A*); OR: 2.1, 95% CI:1.4–3.2, *p* < 0.001 rs7145618 (*PPP2R5C*); OR: 3.1, 95% CI:1.5–6.4), *p* =0.01 rs17722392 (*KIDINS220*); OR: 3.2, 95% CI: 1.5–6.9, *p* = 0.02 rs2265919 (*SHPRH*); OR:2.0, 95% CI: 1.3–3.16, *p* = 0.002 rs1783606 (*SHANK2*); OR:2.4, 95% CI: 1.3–4.1, *p* = 0.02 rs2265477 (*SHPRH*); OR:1.8, 95% CI: 1.2–2.8, *p* = 0.01 rs2069763a (*IL-2*); OR:0.48, 95% CI: 0.3–0.8, *p* = 0.01 rs6903252 (Intergenic); OR:1.8, 95% CI: 1.2–2.8, *p* = 0.01 rs2265917 (*SHPRH*); OR:1.8, 95% CI: 1.2–2.8, *p* = 0.01 rs16936667 (*PRDM14*); OR:2.2, 95% CI: 1.2–3.8, *p* = 0.02 rs341497 (*DIAPH3*); OR:2.6, 95% CI: 1.3–5.2, *p* = 0.004 rs1016429 (*GRIN3A*); OR:2.5, 95% CI: 1.2–5.0, *p* = 0.01 rs10117679 (*GRIN3A*); OR:2.8, 95% CI: 1.2–6.4, *p* = 0.01 rs1871184 (*ITGA1*); OR:1.9, 95% CI: 1.1–3.1, *p* = 0.01 rs3212574 (*ITGA2*); OR:1.7, 95% CI: 1.1–2.7, *p* = 0.01 rs2240747 (*ZNRF4*); OR:1.8, 95% CI: 1.1–3.1, *p* = 0.01
Yao, 2013 [69]	16 cases of PTDM after kidney transplantation and 89 controls.	PTDM was defined as FBG ≥ 125 mg/dL (7.0 mmol/L). Controls have been defined as patients who did not develop PTDM. There is evidence of kidney or diabetic damage among controls.	Patients aged > 18 years who maintained graft function for at least six months with no prior history of diabetes or transplantation. Follow-up duration ≥ six months. The *Fok1* polymorphisms of the *VDR* gene were genotyped for the study.	Fok1 f allele (*LEP*); OR:11.8, 95% CI:1.7–80.1, *p =* 0.012
Ling, 2013 [70]	25 cases of PTDM after liver transplantation and 100 controls.	PTDM was defined according to the ADA criteria: FBG level ≥ 7 mmol/L (126 mg/dL), or a non-FBG ≥ 11.1 mmol/L (200 mg/dL) (at least two occasions) or the requirement for anti-diabetic drugs after the first month of transplantation. Controls have been defined as patients who did not develop PTDM. There is evidence of liver or diabetic damage among controls.	Patients with no previous history of diabetes and with functional graft and more than six months follow-up time. The mean follow-up was 6–61 months. Four SNPs, rs290487, rs7903146, rs11196205, and rs12255372, of the *TCF7L2* gene were genotyped for the study.	rs290487 (*TCF7L2*); OR:1.9, 95% CI:1.1–3.5, *p =* 0.04
Lee, 2013 [71]	49 cases of PTDM after kidney transplantation and 253 controls.	PTDM was defined as continuous FBG levels ≥ 126 mg/dL (7.0 mmol/L); plasma glucose concentrations ≥ 200 mg/dL (11.1 mmol/L); or 2 h post-load glucose ≥ 200 mg/dL (11.1 mmol/L) during an OGTT; or requirement for insulin and/or oral anti-diabetic agents for more than three months. Controls have been defined as patients who did not develop PTDM. There is evidence of kidney or diabetic damage among controls.	Patients with no history of diabetes or severe metabolic or infectious disease before transplantation. The mean follow-up duration was 87.91 months (87.91 ± 78.23 months). *ACE* and *AGT* gene polymorphisms were genotyped for the study.	rs4762 (*AGT*); OR:2.1, 95% CI:1.1–3.8, *p =* 0.01
Kim, 2012 [72]	53 cases of PTDM after kidney transplantation and 253 controls.	PTDM was defined as FPG concentration over 126 mg/dL, HbA1c above 6.5%, or insulin and oral anti-diabetic agents requirement for over three months. Controls have been defined as patients who did not develop PTDM. There is evidence of kidney or diabetic damage among controls.	Patients with no history of diabetes or severe metabolic or infectious disease before transplantation. The mean follow-up duration was 79.45 months. Eighteen SNPs of interleukin were genotyped for the study.	rs1494558 (*IL-7R*); OR:1.6, 95% CI: 1.1–2.5, *p =* 0.03 rs2172749 (*IL-7R*); OR:1.6, 95% CI: 1.1–2.5, *p =* 0.04 rs4819554 (*IL-17R*); OR:1.8, 95% CI: 1.1–2.9, *p =* 0.01 rs2069763 (*IL-2*); OR:1.6, 95% CI: 1.1–2.4, *p =* 0.04
Tavira, 2012 [73]	115 cases of PTDM after kidney or heart transplantation and 205 controls.	PTDM was defined based on the WHO guidelines: FBG > 125 mg/dL (7.0 mmol/L) after three consecutive measurements. Controls have been defined as patients who did not develop PTDM. There is no evidence of kidney or diabetic damage among controls.	Patients > 18 years with no history of diabetes before transplantation. Follow-up period for one year. SNPs rs5219 and rs1805127 of *KCNJ11* were genotyped for the study.	rs5219 (*KCNJ11*); OR:2.1, 95% CI: 1.2–3.5, *p =* 0.004
Tavira, 2011 [47]	145 cases of PTDM after kidney transplantation and 260 controls.	PTDM was defined as FBG > 125 mg/dL after three consecutive measurements) in the first year post-transplant. Control patients were defined as patients who remained non-diabetics during the first year post-transplant. There is evidence of kidney or diabetic damage among controls.	Caucasian patients > 18 years with no history of diabetes before transplantation. Follow-up period for one year. SNPs rs2237895 (A/C) and rs2237892 (C/T) in intron 15 of *KCNQ1* and rs8234 in the 3′ untranslated region (UTR) were genotyped for the study.	rs2237895 (*KCNQ1*); OR:1.83, 95% CI: 1.4–2.9, *p* = 0.008
Yang, 2011 [74]	133 cases of PTDM after kidney transplantation and 170 controls.	PTDM was defined as having FBG level of more than 126 mg/dL one month or later after transplantation (at least two occasions) and/or the requirement for anti-diabetic drugs during the follow-up. Controls have been defined as patients who did not develop PTDM. There is evidence of kidney or diabetic damage among controls.	Hispanic patients with no history of diabetes and organ transplant before transplantation. Follow-up period of at least one year. SNPs of *TCF7L2*, *HNF4A*, *HNF1A*, *KCNJ11*, *SUR1*, *ENPP1*, *PPARG*, *PPARGC1*, and *IRS1* were genotyped for the study.	rs2144908 (*HNF4A*); OR:1.9, 95% CI: 1.1–3.5, *p =* 0.01 rs1884614 (*HNF4A*); OR:2.4, 95% CI: 1.4–4.4, *p =* 0.002 rs1801278 (*IRS1*); OR:2.7, 95% CI: 1.2–6.9, *p =* 0.02 rs5219 (*KCNJ11*); OR:1.5, 95% CI: 1.1–2.1, *p =* 0.01
Kurzawski, 2011 [75]	66 cases of PTDM after kidney transplantation and 168 controls.	PTDM was defined as HgA1c levels continuously over 6.5 mg/dL, FBG levels over 126 mg/dL, or the requirement for insulin and/or oral anti-diabetic agents for over three months. Controls have been defined as patients who did not develop PTDM. There is evidence of kidney or diabetic damage among controls.	Caucasian patients with no history of diabetes with functioning graft for at least one year. Follow-up period for one year. SNPs rs7903146 and rs12255372 of the *TCF7L2* gene were genotyped for the study.	rs7903146 (*TCF7L2*); OR:4.1, 95% CI: 1.2–14.33, *p =* 0.01
Jeong, 2010 [76]	56 cases of PTDM after kidney transplantation and 255 controls.	PTDM was defined as continuous hemoglobin A1c levels over 6.5%, FBG concentration over 126 mg/dL, or insulin and/or oral anti-diabetic agents required for over three months. Controls have been defined as patients who did not develop PTDM. There is evidence of kidney or diabetic damage among controls.	Patients with no history of diabetes or severe metabolic or infectious disease before transplantation. The mean follow-up duration was 89.62 months. Three SNPs (rs2107538 in promoter-403, rs2280789 in intron_1, and rs3817655 in intron_2) of the *CCL5* gene were genotyped for the study.	rs2107538 (*CCL5*); OR:1.8, 95% CI: 1.2–2.8, *p =* 0.003 rs2280789 (*CCL5*); OR:1.7, 95% CI: 1.2–2.6, *p =* 0.01 rs3817655 (*CCL5*); OR:1.8, 95% CI: 1.2–2.8, *p* = 0.003
Kang, 2008 [77]	119 cases of PTDM after kidney transplantation and 392 controls.	PTDM was defined according to ADA criteria: patients required anti-diabetic drugs or insult after the third post-transplantation month. Controls were a combination of patients who did not develop PTDM and patients who developed diabetes during the first year following transplantation but recovered to normoglycemia without medication. There is no evidence of kidney or diabetic damage among controls.	Patients with no history of diabetes, severe metabolic or infectious disease, and organ transplant before transplantation. Follow-up period for one year. SNPs rs4506565, rs7901695, rs7903146, rs11196205, rs12243326, and rs12255372 of the *TCF7L2* gene were genotyped for the study.	rs7903146 (*TCF7L2*); OR:2.7, 95% CI: 1.3–6.2, *p =* 0.01
